# Supplementary material for: Explainable classification of goat vocalizations using convolutional neural networks
Source: PLoS One. 2025 Apr 1;20(4):e0318543. doi: 10.1371/journal.pone.0318543 (PMC11960873; doi:10.1371/journal.pone.0318543)
Supplement: Attachment — Submitted filename: ReviewResponseLetter.pdf [file pone.0318543.s001.pdf]

Article ID: PONE-D-24-24074
Title: "Explainable Classification of Goat Vocalizations Using Convolutional Neural Networks"
Authors: Stavros Ntalampiras and Gabriele Pesando Gamacchio

Authors' response to the comments and suggestions made by the Editor and the Reviewers

All review comments were considered and incorporated into the updated manuscript. For your convenience, we provide the review comments in italics, followed by the authors' response.

Comments from the Editor:
Thank you for submitting your manuscript to PLOS ONE. After careful consideration, we feel that it has merit but does not fully meet PLOS ONE's publication criteria as it currently stands. Therefore, we invite you to submit a revised version of the manuscript that addresses the points raised during the review process.

Response of the author:
Thank you very much for the opportunity to improve the paper. All comments were implemented as you see in the following pages. We would like to thank the Editor and the Reviewers for their constructive comments and suggestions.

Best regards,
Stavros Ntalampiras and Gabriele Pesando Gamacchio

1. Response to the Feedback received from the Academic Editor

Comment from the Editor:
1) Please describe the class labels i.e. [heat, feed distribution, parturition, injury/death, social isolation, simultaneous presence of mothers and kids, presence of unknown visitors, and mother-kid separation] so that the current research helps future research to be built upon it. It will be helpful if the rationale for choosing these labels is provided. Please clarify, if heat is used as a label, what temperature is considered for the label to be considered "heat"? Can two labels exist simultaneously, for example, can "heat" exist with "social isolation"? If so, which label is assigned to the recording in this case?

Response of the author:
Thank you very much for your comment. To address it we added a description of each class of goat vocalizations:

- on page 4, line 100, the text now is:

"The VOCAPRA dataset encompasses a diverse array of emotional states experienced by goats in response to specific stimuli and situations encountered in their daily lives. These states are categorized into eight distinct classes:

Heat: Reflecting the physiological and behavioral changes associated with reproductive cycles and mating.

Feed Distribution: Capturing vocalizations elicited during feeding times, shedding light on social dynamics and resource allocation within goat herds.

Injury or Death: Documenting vocal expressions in response to injuries or mortality events, offering insights into distress signals and herd reactions to adverse events.

Social Isolation: Unveiling vocal cues associated with separation from herd members, highlighting the significance of social bonds and group cohesion among goats.

Mother-Kid Separation: Observing vocalizations arising from the separation of mother goats and their offspring, elucidating maternal instincts and bonding dynamics.

Parturition: Documenting vocalizations during the birthing process, providing crucial insights into maternal behaviors and birthing rituals among goats.

Presence of Unknown Visitors: Recording responses to unfamiliar human or animal presence, unveiling vigilance and territorial behaviors in farm settings.

Mother-Kid Reunion: Capturing vocal interactions upon reunion between mother goats and their kids, illuminating the significance of maternal bonds and social reunification in goat herds."

In addition, as mentioned in the Problem Formulation section this work assumes that at each time instance, there is one dominating audio class, which is rather reasonable since the above-mentioned vocalizations hardly ever occur together [22].

Comment from the Editor:
2) Please mention the version of MobileNet that was used for their experiments since multiple versions of MobileNet are available. See: https://keras.io/api/applications/mobilenet/

Response of the author:
Thank you very much for detecting this omission. Please note that we used the second version of MobileNet and this piece of information was added to the paper as follows:

- on page 8, line 241, the text now is:

"To expedite the classification process, we opted for transfer learning using the pretrained MobileNet V2, leveraging its initial weights trained on the ImageNet dataset."

Furthermore, the implementation is now publicly available at https://colab.research.google.com/drive/1Xu-U3HY_FHgI11Ni35I69DdlP7jFFOLD#scrollTo=AMb1H9dgFsNp to enable a full reproduction of the entire experimental pipeline favouring open science practises.

Comment from the Editor:
3) Please discuss and confirm that the proposed CNN architecture, EfficientNetB0, and MobileNet models were trained with the training/testing setup i.e. maximum number of epochs, learning rate, early stopping, batch size, the same seed for cross-validation etc. For the sake of balanced comparison, the same experimental setup should be used. If this is not the case, please provide adequate justification?

Response of the authors:
Thank you for this comment. Please note that we followed the same experimental protocol across all considered models (proposed CNN architecture, EfficientNetB0, and MobileNet) to ensure a reliable comparison. The model training parameters (number of epochs, learning rate, early stopping, and batch size) were optimized for each architecture. At the same time, we used stratified 5-fold cross-validation to divide the available dataset. All these aspects are now more detailed in section "Experimental Results and Analysis". Moreover,

- on page 7, line 226, the text now is:

"To address class imbalance, we adopted a stratified 5-fold cross-validation scheme. As regards to the overfitting phenomenon, we monitored training and validation accuracy as well as the respective losses, where we confirmed a steady mutual increase indicating improved performance after each iteration."

Comment from the Editor:
4) Given the class imbalance from Figure 3, one would think that macro-averaged f1-score is the more suitable metric to measure classification performance. In light of this, can the authors justify the use of classification accuracy as opposed to f1-score as the metric in their experiments?

Response of the authors:
Thank you for this comment. Please note that class imbalance did not affect the test set since we used stratified 5-fold cross-validation. Furthermore, model testing was carried out only on original data without considering their augmented versions \cite{Shorten2019}.

- on page 6, line 173, the text now is:

"The augmentation parameters were selected ensuring that the identity of the goat vocalization class would not be altered up to the extent that a human listener can assess. It should be mentioned that model testing is carried out only on original data without considering their augmented versions \cite{Shorten2019}."

Comment from the Editor:
5) In light of feedback from Reviewer 2, it is recommended that the authors provide source code to reproduce their experimental results and bring necessary context to the classification of goat vocalizations.

Response of the authors:
Thank you for this comment. We addressed the comments of Reviewer 2 as you may see below. Furthermore, the implementation is now publicly available at https://colab.research.google.com/drive/1Xu-U3HY_FHgI11Ni35I69DdlP7jFFOLD#scrollTo=AMb1H9dgFsNp to enable a full reproduction of the entire experimental pipeline.

Comment from the Editor:
6,7) Please address the feedback by Reviewer 2 regarding preprocessing artifacts being identified as relevant features in your experiments. 7. It will be useful if the contribution of this experimental work is clarified in light of reviewer feedback.

Response of the authors:
Thank you for this comment. We addressed the comments of Reviewer 2 as you may see below. More precisely, we fully re-ran the entire experiment and made sure that the operation (training, validation and testing) of the proposed model is not affected by the audio signal preprocessing phase.

1. Response to the Review 1

Comment from the Reviewer:

1) The paper titled "Explainable Classification of Goat Vocalizations Using Convolutional Neural Networks" is a wonderful attempt to focus on sounds produced by goats in different circumstances. However it claims that automatic monitoring of goat farms is not available in literature which should be written as to the best of our knowledge the automatic monitoring of goat farms is unavailable in literature and it also boasts about it being the first time automatic classification of goat vocalizations is addressed. How could this claim be justified?

Response of the authors:

Thank you very much for your kind criticism. Indeed, to the best of knowledge, the automatic acoustic monitoring of goat farms has not been systematically explored in the literature. Following the comment, the specific sentence was rephrased as follows:

- on page 2, line 21, the text now is:

"Unfortunately, to the best of our knowledge, automatic acoustic monitoring of goat farms has not been systematically explored in the related literature."

Comment from the Reviewer:

2) The diversity of the vocapra dataset in terms of the number of farms, breeds, and environmental conditions needs to be further highlighted. The parameters for pitch shifting and time stretching selected needs to be elaborated and further what other data augmentation techniques were available and the reasons for not using them if any.

Response of the authors:

Thank you very much for this comment. Please note that there is another paper detailing the VOCAPRA dataset where the diversity of the dataset is highlighted [1]. To address the comment, we added a description of each class of goat vocalizations:

- on page 4, line 100, the text now is:

"The VOCAPRA dataset encompasses a diverse array of emotional states experienced by goats in response to specific stimuli and situations encountered in their daily lives. These states are categorized into eight distinct classes:

Heat: Reflecting the physiological and behavioral changes associated with reproductive cycles and mating

Feed Distribution: Capturing vocalizations elicited during feeding times, shedding light on social dynamics and resource allocation within goat herds.

Injury or Death: Documenting vocal expressions in response to injuries or mortality events, offering insights into distress signals and herd reactions to adverse events.

Social Isolation: Unveiling vocal cues associated with separation from herd members, highlighting the significance of social bonds and group cohesion among goats.

Mother-Kid Separation: Observing vocalizations arising from the separation of mother goats and their offspring, elucidating maternal instincts and bonding dynamics.

Parturition: Documenting vocalizations during the birthing process, providing crucial insights into maternal behaviors and birthing rituals among goats.

Presence of Unknown Visitors: Recording responses to unfamiliar human or animal presence, unveiling vigilance and territorial behaviors in farm settings.

Mother-Kid Reunion: Capturing vocal interactions upon reunion between mother goats and their kids, illuminating the significance of maternal bonds and social reunification in goat herds."

As regards to data augmentation, pitch shifting and time stretching were selected as they comprise well-established choices in the audio pattern recognition literature [2]. The augmentation parameters were selected based on listening tests with the aim being augmenting the available data while maintaining the class id. Importantly, the test set includes only original data without any type of augmentation. This information was included in the paper as follows:

- on page 6, line 173, the text now is:

"The augmentation parameters were selected ensuring that the identity of the goat vocalization class would not be altered up to the extent that a human listener can assess. It should be mentioned that model testing is carried out only on original data without considering their augmented versions \cite{Shorten2019}."

[1] Ntalampiras S, Ludovico LA, Presti G, Vena MV, Fantini D, Ogel T, et al. An integrated system for the acoustic monitoring of goat farms. Ecological Informatics. 2023;75:102043. doi:10.1016/j.ecoinf.2023.102043.
[2] Salamon J, Bello JP. Deep convolutional neural networks and data augmentation for environmental sound classification. IEEE Signal processing letters. 2017;24(3):279–283.

Comment from the Reviewer:

3) The training and validation graphs could have been added to show how much reliable the model is if possible. In selection of classes a very important class "Predator" should have been added and analyzed. Overall the paper is a nice contribution where authors have put forward the ideas and thoughts in a reasonable way.

Response of the authors:

Thank you very much for this comment. Please note that the training and validation graphs can be extracted using the code provided in the implementation page available at https://colab.research.google.com/drive/1Xu-U3HY_FHgI11Ni35I69DdlP7jFFOLD#scrollTo=AMb1H9dgFsNp. Unfortunately, the class predator is not included in the VOCAPRA database even though it would be interesting to process such data as well. Thank you very much for your encouraging feedback. We are currently working on the points included in the future work.

2.Response to the Review 2

Comment from the Reviewer:

1) The abstract is vague and short on specific statements. What does it mean that your results are "notably accurate"? (instead, provide quantitative metrics such as precision and recall). What were the results of the "detailed analysis", did you identify any specific time-frequency content that was relevant? It then mentions a "proposed framework" in the second to last sentence that wasn't mentioned elsewhere, which "encompasses an interaction scheme" (what does it mean to encompass it, and what is the interaction scheme?). Concrete details rather than vague generalizations will make the abstract much more useful.

Response of the authors:

Thank you very much for this comment. The abstract wase rewritten as follows to specify these aspects and address the comment:

"Efficient precision livestock farming relies on having timely access to data and information that accurately describes both the animals and their surrounding environment. This paper advances classification of goat vocalizations leveraging a publicly available dataset recorded at diverse farms breeding different species. We developed a Convolutional Neural Network (CNN) architecture tailored for classifying goat vocalizations, yielding an average classification rate of 95.8\% in discriminating various goat emotional states. To this end, we suitably augmented the existing dataset using pitch shifting and time stretching techniques boosting the robustness of the trained model. After thoroughly demonstrating the superiority of the designed architecture over the contrasting approaches, we provide insights into the underlying mechanisms governing the proposed CNN by carrying out an extensive interpretation study. More specifically, we conducted an explainability analysis to identify the time-frequency content within goat vocalisations that significantly impacts the classification process. Such an XAI-driven validation not only provides transparency in the decision-making process of the CNN model but also sheds light on the acoustic features crucial for distinguishing the considered classes. Last but not least, the proposed solution encompasses an interactive scheme able to provide valuable information to animal scientists regarding the analysis performed by the model highlighting the distinctive components of the considered goat vocalizations. Our findings underline the effectiveness of data augmentation techniques in bolstering classification accuracy and highlight the significance of leveraging XAI methodologies for validating and interpreting complex machine learning models applied to animal vocalizations."

Comment from the Reviewer:

2) L75-83: what are these classes? It seems they are supposed to represent some sort of behavioral state of the goats, but they also seem like an eclectic (perhaps incomplete) and non-mutually-exclusive set of emotional states. Are there other states that you ignore? Can you justify or explain how they were chosen, and whether there is any existing literature on each of these behavioral states corresponding to specific vocalization qualities? Also, how do you determine the "true" state of the goat? Is there a "none" or "other" category for when none of these classes are dominant? Also, a minimal description of the meaning of these states is warranted - in particular, I don't know what "feed distribution" or "parturition" mean.

Please explain the acoustic data collection of the annotated dataset: are recordings taken opportunistically with a handheld recorder? Extracted randomly, systematically, or haphazardly from continuous audio recordings? How are they trimmed and post-processed? I would encourage the authors to focus on the novel application of these techniques to an interesting problem of within-species variation associated with behavioral states.

Response of the authors:

Thank you very much for this comment. Please note that the recordings are not eclectic; as mentioned in the paper, they are the result of 1-year long continuous 24/7 monitoring of 4 different goat farms using a wireless acoustic sensor network. The recorded vocalizations were annotated by experienced animal scientists. Please note that an exhaustive description of the dataset falls outside the scope of this work since it is already described in [1]. To clarify the content of each class, we added the respective description:

- on page 4, line 100, the text now is:

"The VOCAPRA dataset encompasses a diverse array of emotional states experienced by goats in response to specific stimuli and situations encountered in their daily lives. These states are categorized into eight distinct classes:

Heat: Reflecting the physiological and behavioral changes associated with reproductive cycles and mating

Feed Distribution: Capturing vocalizations elicited during feeding times, shedding light on social dynamics and resource allocation within goat herds.

Injury or Death: Documenting vocal expressions in response to injuries or mortality events, offering insights into distress signals and herd reactions to adverse events.

Social Isolation: Unveiling vocal cues associated with separation from herd members, highlighting the significance of social bonds and group cohesion among goats.

Mother-Kid Separation: Observing vocalizations arising from the separation of mother goats and their offspring, elucidating maternal instincts and bonding dynamics.

Parturition: Documenting vocalizations during the birthing process, providing crucial insights into maternal behaviors and birthing rituals among goats.

Presence of Unknown Visitors: Recording responses to unfamiliar human or animal presence, unveiling vigilance and territorial behaviors in farm settings.

Mother-Kid Reunion: Capturing vocal interactions upon reunion between mother goats and their kids, illuminating the significance of maternal bonds and social reunification in goat herds.

By delineating these distinct emotional states and situational contexts, the VOCAPRA dataset serves as a valuable resource for researchers seeking to unravel the complexities of goat communication and behavior in diverse farming environments.

For more information regarding the recording campaign and dataset composition, the interested reader is referred to \cite{Ntalampiras2023}."

We agree that it would be interesting to investigate the within-species variation along with the associated behavioral states, and was added to the future work section. The first step would be to classify the behavioral states in goat vocalization which is now missing from the literature.

[1] Ntalampiras S, Ludovico LA, Presti G, Vena MV, Fantini D, Ogel T, et al. An integrated system for the acoustic monitoring of goat farms. Ecological Informatics. 2023;75:102043. doi:10.1016/j.ecoinf.2023.102043.

Comment from the Reviewer:
3) The comparison of existing and proposed model architectures is undermined by training them in different ways (different batch size, different number of epochs and stopping procedure). Why were they trained for different numbers of epochs? If you use early stopping on the existing architectures, are they perhaps less over-fit and do they perform better on the test data? Did you use suturing performance on the validation set to determine when to stop training the existing as well as proposed architectures? Additionally, it is unclear whether the "transfer learning" approach trains the full network or only the added final layers (see minor comment below) - these two scenarios fundamentally change the interpretation of the results, and training the full network is certainly necessary to test the question posed (which architecture is best?).

Response of the authors:
Thank you very much for this comment. Please note that model architectures were training in a standardized way adopting early stopping criterion by monitoring the validation loss. The training parameters were optimised according to the characteristics of each architecture. Please note that the training and validation graphs can be extracted using the implementation which is available at https://colab.research.google.com/drive/1Xu-U3HY_FHgI11Ni35I69DdlP7jFFOLD#scrollTo=AMb1H9dgFsNp. Also, please note that the transfer learning approach retrains only the added layers, which is a well-established process in the literature and does not undermine model comparison. This is clarified in the following text:

- on page 8, line 240, the text now is:

"5.1.1 MobileNet
To expedite the classification process, we opted for transfer learning using the pretrained MobileNet V2, leveraging its initial weights trained on the ImageNet dataset. This approach benefits from the knowledge captured by MobileNet while avoiding the need to train the neural network from scratch. In addition to the pre-trained layers, we added two dense layers and a final classification layer to fine-tune the network for the task at hand.

5.1.2 EfficientNetB0
Following an analogous line of thought, we added two dense layers with dimension 4096."

Comment from the Reviewer:

4) It is imperative to test the generalizability of models by testing them on held-out data that is not simply a random subset of the training and validation data. It is unclear in the manuscript whether the cross-validation test datasets are random splits from the training and validation data, or whether an entire data source (e.g. a separate farm) is used as a testing set for each "fold". The latter will provide much stronger evidence of the model's ability to generalize beyond the training samples.

Response of the authors:
Thank you very much for this comment. We agree that it would be interesting to test the models on data coming from different farms, conditions, etc. but such information is not available in the specific dataset. Nonetheless, the fact that the proposed model achieves excellent 95.8\% accuracy using a stratified 5-fold cross-validation experimental protocol is more than encouraging. These considerations are discussed in the end of Section 5.2.

Comment from the Reviewer:
5) I think the CRP approach is valuable here for interpreting the model's attention to various details, I interpret the results differently from the authors' interpretation. Specifically, the CRP is uncovering that the custom model is "cheating" by using some quirk of the preprocessed audio for classification, that is not actually relevant to the goats' vocalizations.

Response of the authors:
Thank you very much for this comment. Please note that the feature extraction and normalization procedures are common for all classes and no class-specific processing was carried out. However, we understand how Fig. 1 can be misleading given the upper black area. After carefully examining the dataset, we removed the high-frequency bands where no vocalization content is present, while the values are practically equal to zero and re-run the entire experimental phase. Importantly, the classification results did not change significantly, while the interpretation analysis was updated to reflect the updated model.

As a result, we updated the following parts of the paper: Figures: 1, 5, 6, 7, and 8 and Sections 5.2 and 6 on pages 8-13.

Comment from the Reviewer:
6) L14-30 could you motivate the idea of studying goat vocalizations for animal welfare monitoring by first providing some literature/evidence that goat's vocalizations encode their emotional state?

Response of the authors:
Thank you very much for this comment. To support the specific claim, one more reference was added in the specific text.

- on page 2, line 29, the text now is:

"Interestingly, the authors of [19] showed that goats are able to distinguish between positive and negative emotion-linked vocalizations, thus they may be used for animal welfare monitoring [20]."

Comment from the Reviewer:
7) L30-35: This paragraph doesn't say what kind of data the VOCAPRA project collects: recordings of individual goats? Association of specific vocalizations with behaviors? Recordings of groups of goats? Annotations of the audio recordings? Please provide some relevant details on how the data is useful for the task at hand.

Response of the authors:
Thank you very much for this comment. More information was added to describe the VOCAPRA dataset. Please note that more information on the dataset is available in the given reference.

- on page 2, line 32, the text now is:

"The above-mentioned literature gap may be attributed to the limited availability of a thorough dataset of goat vocalization since publicly-available datasets are practically non-existent. To cover this gap, the VOCAPRA project[1] implemented such a dataset with the ultimate goal being the real-time provision of data, information and insights to farmers, animal scientists, technicians, etc. The VOCAPRA project has collected a valuable dataset of goat vocalizations from different farms located in Lombardy, Italy. The dataset includes recordings of both individual and groups of goats which have been annotated by experienced animal scientists as detailed in [23]."

Comment from the Reviewer:

8) L39: "the problem at hand" is vague, what is the CNN designed to do? Is it a supervised classification task or some other kind of task (eg unsupervised clustering)? What are the classes or clusters it should be able to distinguish?

Response of the authors:
Thank you very much for this comment. To address the comment, the specific text was rephrased as follows:

- on page 2, line 42, the text now is:

"design and implementation of a Convolutional Neural Network (CNN) tailored to the classification of goat vocalizations,"

Please note that the classes are described in the section entitled "The VOCAPRA dataset".

Comment from the Reviewer:
8) L41: I don't know what you mean by a "standardized feature set" without some context
L43: specify that this the augmentation is for training the CNNs

Response of the authors:
Thank you very much for this comment. Please note that the entire phrase reads "utilization of a standardized feature set eliminating the necessity of domain expertise," thus standardize means that it is free of domain knowledge. Regarding the augmentation, the phrase was updated as follows:

- on page 3, line 46, the text now is:

"consideration and application of suitable data augmentation techniques during training the CNNs"

Comment from the Reviewer:
9) L56: please provide a high-level description of what Concept Relevance Propagation does, and how itis useful to XAI.

Response of the authors:
Thank you very much for this comment. The following sentence was added to describe the CRP. More information is given in the respective section

- on page 3, line 62, the text now is:

"CRP extends the functionality of back-propagation by introducing the concept of conditions enabling the identification of particular channels and layers that have a significant impact on the final prediction."

Comment from the Reviewer:
10) Fig 3: the classes don't seem to map 1:1 onto the list given in L76, please clarify.

Response of the authors:
Thank you very much for this comment. It is not clear which class(es) is(are) missing from the Figure. All the classes described in Section 3 are included in Fig. 3 (the names have been shortened to favour visualization).

Comment from the Reviewer:
11) L125: in contrast to what? Providing the alternative (some sort of manual feature specification) would make this point more clear.

Response of the authors:
Thank you very much for this comment. Handcrafted feature examples may be the class-specific feature design such as spectral roll-off, band-specific energies, etc and in general everything which is tailored to processing specific sound classes. To clarify this point, the sentence was rephrased as follows:

- on page 6, line 152, the text now is:

"Unlike handcrafted features, such a standardized feature extraction process minimizes the need for domain expertise for characterizing the available goat vocalizations."

Comment from the Reviewer:
12) L130-133: you could mention the large number of model parameters, which is the typical explanation for why deep learning models are prone to over-fitting.

L167-173: these days CNNs can accommodate various input shapes, and also empirically, rescaling the input sample to different sizes has little effect on classifier performance.

Response of the authors:
Thank you very much for this comment. Even though this part is not refereeing to overfitting, it was updated as proposed to address the comment:

- on page 6, line 157, the text now is:

"CNNs, characterized by their substantial model capacity and large number of parameters, heavily rely on access to extensive training data. This dependency is crucial for effectively learning a non-linear function from input to output, facilitating generalization and achieving high classification accuracy on previously unseen data."

It is true that rescaling the input may not influence the CNN's performance, but it alters the time-frequency content and may result in loss of information of even form artificial content not representative of the addressed problem.

Comment from the Reviewer:
13) How were the training, validation, and testing datasets created? Are they from independent or overlapping sources? This has major implications for the interpretation of model performance. For instance, if testing samples are from the same source as training and validation, the model might be over-fit to the training data but still perform well on the testing data. An ideal testing set is not just a split from k-fold cross validation, but instead a truly separate set of data (e.g. data from a different set of individual goats or a different farm).

Response of the authors:
Thank you very much for this comment. Indeed, the formation of training, validation and testing sets is of paramount importance for model learning and evaluation. As mentioned in the beginning of section 5, we employed a stratified 5-fold cross validation protocol, which is a well-established protocol in the ML community. As mentioned above, farm-specific information is not available in the specific dataset. However, the achieved performance is more than promising and demonstrates that the model is able to generalize over unseen data. The dataset includes a plethora of vocalizations of hundreds of animals recorded at diverse locations.

Comment from the Reviewer:
14) L189: did you initialize this proposed novel architecture with random weights?

Response of the authors:

Thank you very much for this comment. The proposed architecture was indeed initialized with random weights. All layers of the proposed novel architecture were initialized using the default Keras initializers, specifically GlorotUniform for the dense and convolutional layers. No pre-trained weights or transfer learning methods were used in the experiments for this architecture.

Comment from the Reviewer:
15) L210-216: It is unclear here if you only trained the added layers and kept the feature extractor frozen with the imagenet pretrained weights, or trained the entire network. For comparison to your custom architecture, it would be critical to train the entire network. If only training the final layers, you are asking a very different question ("can we perform transfer learning without fine-tuning the feature extractor?", rather than "which architecture is better?"), and it would not be surprising at all to see worse performance when using the frozen feature extractor.

Response of the authors:
Thank you very much for this comment. As mentioned in sections 5.1.1 and 5.1.2 we employed a classical transfer learning methodology dictating the pretrained networks to be used as feature extractors, while adding layers serving the present classification task. Please note that this is the typical way transfer learning technologies are applied. In the present case, it would be impossible to retrain such deep networks on the present dataset which is much smaller in size with respect to their original training sets (e.g. imagenet) without overfitting. This is the typical way as found in the related literature [1].

[1] Ntalampiras, S. (2021). Transfer Learning for Generalized Audio Signal Processing. In: Miranda, E.R. (eds) Handbook of Artificial Intelligence for Music. Springer, Cham. https://doi.org/10.1007/978-3-030-72116-9_23

Comment from the Reviewer:
16) L265: the ability to apply this model beyond the studied system is unclear to me: (1) as mentioned above, it is not clear if your testing set was from a separate location compared to your training and validation set. If not, you have very little idea of how the model will generalize beyond the training data. (2) even if your testing set comes from separate locations compared to the training set, you should discuss the challenges of domain transfer here:

applying the model to new locations/farms/scenarios will likely result in decreased performance depending on the similarity of the soundscape to the original training and testing data.

Response of the authors:
Thank you very much for this comment. Indeed, the limitations of the proposed model can be clarified in the specific part. To implement this comment, the specific text was updated as follows:

- on page 10, line 296, the text now is:

"We argue that the rates achieved by the proposed model are more than satisfactory and highlight the efficacy of the implemented architecture. The proposed model may be efficiently employed to serve precision livestock farming applications \cite{Ntalampiras2023}. However, applying the existing model to unseen locations/farms/scenarios may result in decreased performance depending on the similarity of the soundscape to the original training and testing data. As such, model finetuning and retraining using additional data representing the unknown setting should be carried out."
