# Supplementary material for: Explainable classification of goat vocalizations using convolutional neural networks
Source: PLoS One. 2025 Apr 1;20(4):e0318543. doi: 10.1371/journal.pone.0318543 (PMC11960873; doi:10.1371/journal.pone.0318543)
Supplement: Attachment — Submitted filename: ReviewResponseLetter_auresp_2.pdf [file pone.0318543.s002.pdf]

Article ID: PONE-D-24-24074.R1
Title: "Explainable Classification of Goat Vocalizations Using Convolutional Neural Networks"
Authors: Stavros Ntalampiras and Gabriele Pesando Gamacchio

Authors' response to the comments and suggestions made by the Editor and the Reviewers

All review comments were considered and incorporated into the updated manuscript. For your convenience, we provide the review comments in italics, followed by the authors' response.

Comments from the Editor:
Thank you for submitting your manuscript to PLOS ONE. After careful consideration, we feel that it has merit but does not fully meet PLOS ONE's publication criteria as it currently stands. Therefore, we invite you to submit a revised version of the manuscript that addresses the points raised during the review process.

Response of the author:
Thank you very much for the opportunity to improve the paper. All comments were implemented as you see in the following pages. We would like to thank the Editor and the Reviewers for their constructive comments and suggestions.

Best regards,
Stavros Ntalampiras and Gabriele Pesando Gamacchio

1. Response to the Feedback received from the Academic Editor

Comment from the Editor:

1) The comment regarding the Training, Validation, and Test set divisions. If experiment cannot be performed at this moment, as per the suggestion of the reviewer, it may be worth adding that experiment as a limitation or future work while providing a reasonable justification why that experiment was not performed in the current manuscript.

Response of the authors:

Thank you very much for your comment. It is true that audio data can be highly auto-correlated but please note that we did not split any vocalization file during our analysis. As such, the case of having vocalizations from the same file into training and testing set is not possible. At the same time, information whether different files were recorded during the same scenario is not available; however, this does not necessarily lead to overfitting since there may be multiple goats vocalizing in the same scenario, hence different vocalizations may come from different individuals/groups. As mentioned in the paper, the dataset includes recordings of both individual and groups of goats which have been annotated by experienced animal scientists.

With respect to the geographical location, as mentioned in the previous response letter, this information is not included in the dataset. Thus, the present *k*-fold cross validation protocol is the choice which is as close as possible to the real-world conditions offered by the VOCAPRA dataset.

We agree that it would be interesting to test how the model performs across locations/farms/scenarios, but this is not possible at the moment. To address this comment, we included this limitation as follows:

- on page 10, line 296, the text is:

"We argue that the rates achieved by the proposed model are more than satisfactory and highlight the efficacy of the implemented architecture. The proposed model may be efficiently employed to serve precision livestock farming applications \cite{Ntalampiras2023}. However, applying the existing model to unseen locations/farms/scenarios may result in decreased performance depending on the similarity of the overall soundscape to the original training and testing data. As such, model finetuning and retraining using additional data representing the unknown setting should be carried out."

- and on page 15, line 512, the text now is:

"evaluating the performance of the proposed model across locations, farms, etc."

Comment from the Editor:

2) The comment regarding comparison of the "custom CNN" and pretrained models based on ImageNET, and the rationale for proposing a custom architecture. I suppose one may compare in terms of model size and parameter count, but I encourage you to provide a reasonable rationale.

Response of the authors:

Thank you very much for your comment. It was addressed in detail as you see below. The main reason for proposing a custom architecture is that this is the first time that automatic classification of goat vocalizations is addressed in the literature, so there is no architecture to rely on. This fact underlines the novelty of the present article. We actually tried to train from scratch both MobileNet and EfficientNet but they showed strong signs of overfitting since the data size is limited. Please note that these models have been trained on vast amounts of data.

We agree that it would be interesting to train them from scratch to address the present task as long as more data become available. This was added in the article as a future research direction as follows:

- on page 15, line 513, the text now is:

"training from scratch MobileNet and EfficientNet and evaluate them as long as a sufficient amount of data becomes available"

Also, it should be noted that the feature extraction process is the same for all models and involves the extraction of the log-Mel spectrogram as discussed in section 4.1. As such, no model is used as a feature extractor. This is never mentioned in the paper, and it is not clear why the Reviewer assumed this. The comparison was made since, to the best of our knowledge, this is the first time that automatic classification of goat vocalizations is addressed, hence there are no existing approaches to compare to. Both MobileNet and EfficientNet are popular transfer learning architectures in the ML field [1], thus we decided to employ them.

[1] Shin H-k, Park SH, Kim K-w (2020) Inter-floor noise classification using convolutional neural network. PLoS ONE 15(12): e0243758.

2. Response to the Review 2

Comment from the Reviewer:

1) Training, Validation, and Test set divisions: The authors assert that they could not hold out an entire location for a test set, but that their random division of samples into k-fold validation splits represents a strong test of model performance in real world conditions. I respectfully disagree. Audio data tends to be highly auto-correlated. Imagine for instance that training and test samples for a particular scenario were from two adjacent 3-second audio clips where an individual goat repeatedly made the same vocalization. The model could be highly over-fit on training samples and still perform well on the test set, but might perform very poorly on a fresh set of audio recordings from different individual goats or different dates. It should be at least possible to split out entire original audio recordings (which are then split into many fixed-length samples) into the training and testing sets, if they cannot be split out by geographic location.

Response of the authors:

Thank you very much for your comment. It is true that audio data can be highly auto-correlated but please note that we did not split any vocalization file during our analysis. As such, the case of having vocalizations from the same file into training and testing set is not possible. At the same time, information whether different files were recorded during the same scenario is not available; however, this does not necessarily lead to overfitting since there may be multiple goats vocalizing in the same scenario, hence different vocalizations may come from different individuals/groups. As mentioned in the paper, the dataset includes recordings of both individual and groups of goats which have been annotated by experienced animal scientists. As regards to the geographical location, as mentioned in the previous response letter, this information is not included in the dataset.

Thus, the present *k*-fold cross validation protocol is the choice which is as close as possible to the real-world conditions offered by the VOCAPRA dataset.

We agree that it would be interesting to test how the model performs across locations/farms/scenarios, but this is not possible at the moment. To address this comment, we included this limitation as follows

- on page 10, line 296, the text is:

"We argue that the rates achieved by the proposed model are more than satisfactory and highlight the efficacy of the implemented architecture. The proposed model may be efficiently employed to serve precision livestock farming applications \cite{Ntalampiras2023}. However, applying the existing model to unseen locations/farms/scenarios may result in decreased performance depending on the similarity of the overall soundscape to the original training and testing data. As such, model finetuning and retraining using additional data representing the unknown setting should be carried out."

- on page 15, line 512, the text now is:

"evaluating the performance of the proposed model across locations, farms, etc."

Comment from the Reviewer:

2) It is true that it would be difficult to fine-tune an entire architecture based on a small training set, but the authors trained the "custom CNN" on the training set so I don't understand why they couldn't also compare performance to a variation where the feature extractor is also trained (perhaps with a lower learning rate on the feature extractor than on the added layers). The manuscript frames this experiment as a comparison of architectures, rather than a comparison of feature extractors (architecture + data + training procedure). It is fine to compare the performance of "a custom feature extractor trained on our data with architecture A" to "a pre-trained feature extractor trained on ImageNET with architecture B", but this is not a fair comparison of the architectures A and B themselves, nor is it a fair comparison of training the feature extractor on local data versus transfer learning: both variables have changed simultaneously. If the authors do not wish to run further experiments, they should reframe the framing of the methods and interpretation of the results as "we tried a few reasonable things and our custom model worked best" but not as a rigorous test of one architecture or training approach against another.

Response of the authors:

Thank you very much for this comment. We actually tried to retrain completely both EfficientNetB0 and MobileNetV2 but they showed strong signs of overfitting, thus they were used with frozen weights. Such a strategy comprises a standard practise in the ML field [40]. Please note that these models require a vast amount of data (ImageNet) for proper training. The transfer learning choice was also motivated by the significant differences in the inner workings of the considered models.

Model	Training Approach	Model Total Parameters	Added Dense Layer Parameters	Final total Parameters	Training speed
Custom CNN	Trained from scratch	2,359,808	52,697,032	55,056,840	Fastest
EfficientNet-B0	Transfer learning + fine tuning	4,049,571	22,061,064	26,110,635	Slowest
MobileNetV2	Transfer learning + fine tuning	2,257,984	22,061,064	24,319,048	Slowest

Even though the number of parameters is similar across the models (see the Table above), the training speed of the custom CNN is significantly faster compared to the transfer learning approaches with EfficientNetB0 and MobileNetV2. This is because the custom CNN utilizes standard convolutional and fully connected layers, which are computationally straightforward and efficient. In contrast, EfficientNetB0 and MobileNetV2 incorporate complex mechanisms such as depthwise separable convolutions, squeeze-and-excitation blocks, and other computationally demanding architectural optimizations. While these **mechanisms** may improve efficiency and performance during inference, they increase the computational overhead and slow down the training process.

Overall, we did not proceed with the full training of these two models from scratch because:

- The present dataset is of limited size and training EfficientNetB0 and MobileNetV2 from scratch would have required significantly more data and computational resources.
- As such, the primary aim was to explore the effectiveness of **transfer learning**, i.e. testing whether models pre-trained on **ImageNet** could be adapted to classify goat vocalizations represented as spectrograms. We specifically wanted to evaluate if adding and training dense layers was sufficient to capture the relevant features.

However, we recognize that it would be interesting to train them from scratch when more data becomes available. This was included in the paper as a future direction as follows:

- on page 15, line 513, the text now is:

“training from scratch MobileNet and EfficientNet and evaluate them as long as a sufficient amount of data becomes available”

Furthermore, it should be noted that the feature extraction process is the same for all models and involves the extraction of the log-Mel spectrogram as discussed in section 4.1. As such, no model is used as a feature extractor given that they do not process the audio waveform. The present comparison was made since, to the best of our knowledge, this is the first time that automatic classification of goat vocalizations is addressed, hence there are no existing approaches to compare to or architectures to rely on. Both MobileNet and EfficientNet are popular transfer learning architectures in the ML field, thus we decided to employ them. This point is explained in the following part of the paper:

- on page 8, line 235 the text is

“To the best of our knowledge, this is the first time that automatic classification of goat vocalizations is addressed, hence there are no existing approaches to compare to. As such, the proposed CNN was compared to two diverse and popular deep architectures \cite{Shin2020}, namely MobileNet \cite{howard2017mobilenets} and EfficientNet \cite{DBLP:journals/corr/abs-1905-11946}, which are explained next.”

Comment from the Reviewer:
3) L245: Transfer learning approaches may either freeze or continue training the pre-trained model. Please explicitly state that the pre-trained model weights were frozen during training since that is the strategy used here.

Response of the authors:
Thank you very much for this comment. Please note that this information is already mentioned in the following part of the paper.

- on page 8, line 240, the text is:

“5.1.1 MobileNet

To expedite the classification process, we opted for transfer learning using the pretrained MobileNet V2, leveraging its initial weights trained on the ImageNet dataset. This approach benefits from the knowledge captured by MobileNet while avoiding the need to train the neural network from scratch. In addition to the pre-trained layers, we added two dense layers and a final classification layer to fine-tune the network for the task at hand.

5.1.2 EfficientNetB0

Following an analogous line of thought, we added two dense layers with dimension 4096."

Comment from the Reviewer:
3) Spectrogram input shape (L197): I maintain that you can feed any size image without resizing it into the standard PyTorch implementations of CNN architectures. This is not a justification for needing a custom architecture.

Response of the authors:
Thank you very much for this comment. Please note that CNN architectures require inputs of consistent sizes [1]. We are not aware of how the PyTorch implementation operates; one may employ padding, windowing and/or similar techniques but such a process may result in information loss, degradation, etc. [2]. The justification of the need of a custom architecture was explained in detail in the previous comments.

[1] Swarnendu Ghosh and Nibaran Das and Mita Nasipuri, "Reshaping inputs for convolutional neural network: Some common and uncommon methods", *Pattern Recognition*, 93, pages 79-94, 2019 DOI: https://doi.org/10.1016/j.patcog.2019.04.009.
[2] H. Purwins, B. Li, T. Virtanen, J. Schlüter, S. -Y. Chang and T. Sainath, "Deep Learning for Audio Signal Processing," in *IEEE Journal of Selected Topics in Signal Processing*, vol. 13, no. 2, pp. 206-219, May 2019, doi: 10.1109/JSTSP.2019.290870
